# Case Report: Acute Kidney Injury Due to Chronic Milk-Alkali Syndrome in a Patient With Colon Cancer

**DOI:** 10.3389/fmed.2022.834107

**Published:** 2022-02-04

**Authors:** Hyo Jin Lee, Seokho Yoon, Bong-Hoi Choi, Seunghye Lee, Sehyun Jung, Ha Nee Jang, Se-Ho Chang, Hyun-Jung Kim

**Affiliations:** ^1^Department of Internal Medicine, Gyeongsang National University Hospital, Jinju, South Korea; ^2^Department of Nuclear Medicine, Gyeongsang National University Changwon Hospital, Changwon, South Korea; ^3^Institute of Health Sciences, Gyeongsang National University, Jinju, South Korea; ^4^Department of Nuclear Medicine, Gyeongsang National University Hospital, Jinju, South Korea; ^5^Department of Internal Medicine, Gyeongsang National University College of Medicine, Jinju, South Korea

**Keywords:** hypercalcemia, milk-alkali syndrome, Burnett syndrome, paraneoplastic syndrome, metastatic calcification

## Abstract

**Background:**

Common causes of hypercalcemia include primary hyperparathyroidism and paraneoplastic syndrome of malignancy. Because of this, physicians can easily miss extrinsic causes of hypercalcemia such as milk-alkali syndrome in patients with cancer. We successfully treated a case of acute kidney injury due to severe hypercalcemia caused by milk-alkali syndrome due to long-term milk drinking in a patient with colon cancer.

**Case Description:**

A 62-year-old man was referred to nephrology for hypercalcemia and azotemia that was found during preoperative evaluation for colon cancer surgery. The patient had experienced several months of dizziness and anorexia. We started hemodialysis because hypercalcemia and azotemia were not improved despite large amounts of hydration and diuretics. We suspected paraneoplastic syndrome because of concomitant colon cancer and low intact parathyroid hormone (PTH). Renal microcalcifications were observed on ultrasonography. Metastatic calcifications of the lung and stomach were present, but no malignant metastasis appeared on bone scans. There was no evidence of metastatic malignant lesions on chest or abdominal enhanced computed tomography. PTH-related peptide was not detected. Thus, other causes of hypercalcemia beyond malignancy were considered. On history-taking, the patient reported consuming 1,000 to 1,200 mL of milk daily for the prior 3 months. Hypercalcemia was due to chronic milk-alkali syndrome. We advised withdrawal of milk and nutritional pills. Hemodialysis was stopped after 2 weeks since azotemia and hypercalcemia were resolving. Acute kidney injury was improved, and mild hypercalcemia remained when he underwent hemicolectomy after 1 month. Thereafter, serum calcium and creatinine remained normal at discharge and follow-up for 1 year in the outpatient clinic. However, lung calcifications still remained on bone scan after 1 year.

**Conclusions:**

Chronic milk-alkali syndrome is a rare condition resulting from excessive calcium and alkali intake through various routes, like milk, nutritional supplements, and medicines for osteoporosis. Therefore, early management for hypercalcemia should include precise history taking including diet, previous diagnoses, and current medications.

## Introduction

Hypercalcemia has a wide variety of causes including endocrine disorders, paraneoplastic syndrome, exogenous agents, granulomatous disease, immobilization, rhabdomyolysis, William's syndrome, advanced chronic liver disease, and disseminated cytomegalovirus, among others (1). In practice, primary hyperparathyroidism and malignancy cause the majority (80–90%) of hypercalcemia. In total, 65% of such cases are brought about by malignancies like lung cancer or multiple myeloma (2). Because of this, physicians can overlook other possible causes of hypercalcemia including exogenous agents. Milk-alkali syndrome (MAS) has been defined as hypercalcemia related to metabolic alkalosis from high calcium intake and long-term administration of absorbable alkali (3). We successfully treated acute kidney injury due to severe hypercalcemia caused by chronic MAS due to long-term milk drinking in a patient with colon cancer.

## Case Description

A 62-year-old man was referred to nephrology for hypercalcemia and azotemia that was found during preoperative evaluation for colon cancer surgery. He had dizziness and anorexia for several months. His height and weight were 165.5 cm and 65.7 kg (body mass index 24 kg/m^2^). He had been diagnosed with hypertension seven months ago and had taken 5 mg of amlodipine and 80 mg of valsartan. On physical examination, his blood pressure was 115/48 mmHg, pulse rate was 63 beats/min, respiration rate was 20 breaths/min, and body temperature was 36.4°C. Laboratory findings on admission day included hemoglobin 8.2 g/dL, hematocrit 24%, serum protein 5.7 g/dL, serum albumin 3.1 g/dL, serum phosphate 6.7 mg/dL, serum calcium (sCa) 16.10 mg/dL (reference 8.6–10.2), ionized calcium 7.7 mg/dL (reference 4.5–5.3), serum sodium 138.9 mEq/L, serum potassium 4.5 mEq/L, serum chloride 98.7 mEq/L, serum total CO_2_ 32 mEq/L (reference 21–30), blood urea nitrogen (BUN) 68.3 mg/dL (reference 6–20), serum creatinine (sCr) 5.69 mg/dL and urine chloride 35.9 mEq/L. But we were not able to capture the very first blood gas analysis before hydration started. His initial bicarbonate was supposed to be 31 mEq/L that was calculated from the total CO_2_ (4, 5). The next day after large amounts of intravenous hydration with isotonic saline, he had mixed type of respiratory and metabolic alkalosis (pH 7.49 [reference 7.35-7.45], P_v_CO_2_ 34 mmHg, P_v_O_2_ 52 mmHg, ${\text{HCO}}_{3}^{-}$ 26 mEq/L [reference 23–29], serum sodium 137.4 mEq/L, serum potassium 3.3 mEq/L, serum chloride 99.4 mEq/L). He had hypercalciuria with a urine calcium to creatinine ratio of 0.57 (defined by a urine calcium to creatinine ratio >0.20).

**Figure 1:**
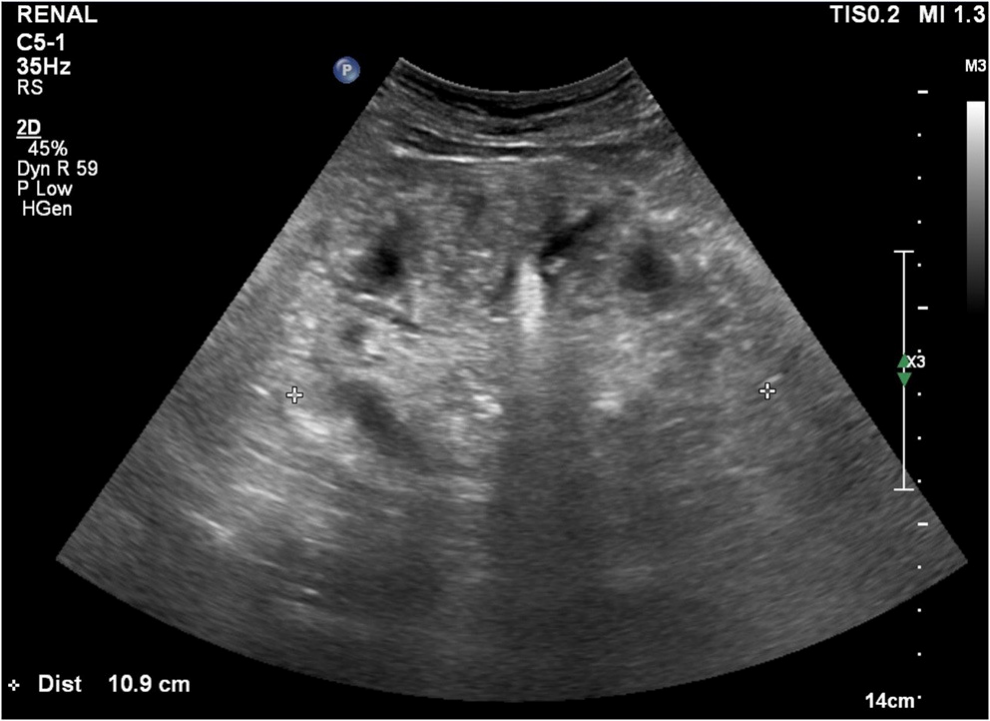
Renal ultrasonography. Multiple renal cortical microcalcifications can be seen.

**Figure 2:**
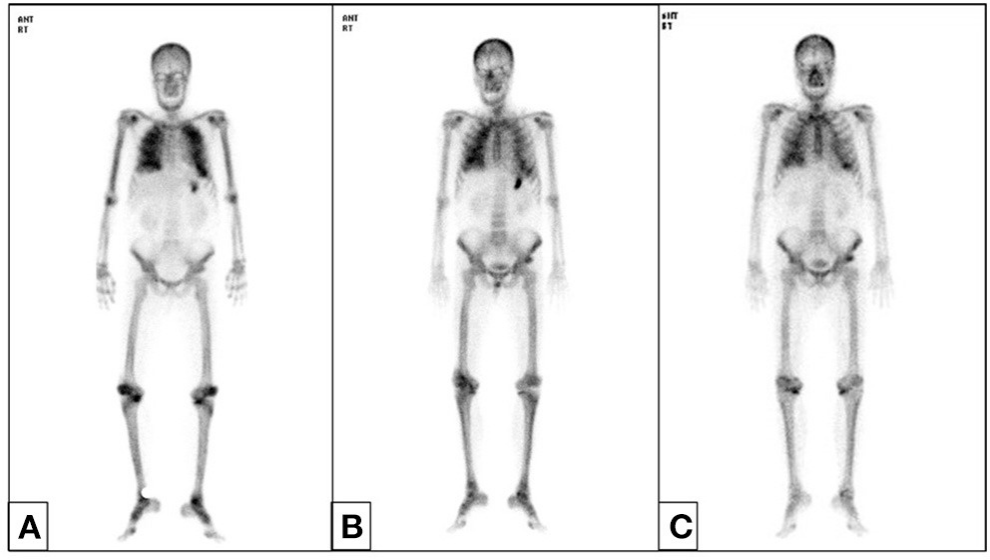
Bone scan. Metastatic calcifications in both lung fields and the stomach appeared without other bone metastatic lesions before hemicolectomy **(A)** and resolved after 6 months **(B)**. Lung calcifications persisted until 1 year **(C)**.

We started hemodialysis with low calcium (2.5 mEq/L) dialysate on admission day 3 because of general weakness, but hypercalcemia and azotemia did not improve despite large amounts of intravenous hydration with isotonic saline and diuretics. We suspected paraneoplastic syndrome due to colon cancer because his intact parathyroid hormone (iPTH) was low (6.01 pg/mL, reference 15–65). Renal microcalcifications were apparent on ultrasonography (Figure 1). Metastatic calcifications in the lung and stomach were observed on bone scans (Figure 2A), but there was no sign of bone metastasis. We did not find any evidence of malignant lesion or granulomatous disease such as sarcoidosis on chest and abdominal enhanced computed tomography (CT) and positron emission tomography (PET)-CT but verified only pneumonia. We prescribed intravenous ceftriaxone and azithromycin for pneumonia due to *Streptococcus pneumoniae* during the admission period. We ruled out PTH-related peptide (PTHrP)-associated hypercalcemia and vitamin D-induced hypercalcemia because PTHrP was not detected (<1.1 *p*mol/L), and 25-OH vitamin D (12.8 ng/ml, reference≥30) and 1,25-(OH)2 vitamin D3 (8.9 pg/mL, reference 19.6–54.3) were low. The patient had osteopenia on bone mineral densitometry (T-score in spine −2.4, left femoral neck −2.1, and right femoral neck −1.8).

**Figure 3:**
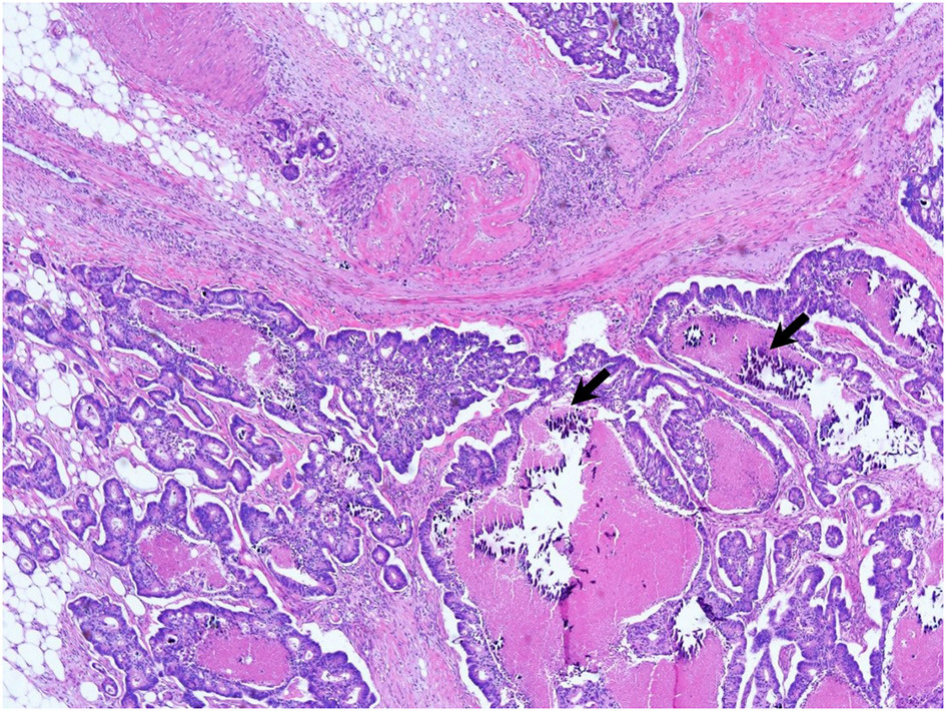
Ascending colon biopsy. Moderately differentiated adenocarcinoma in the ascending colon with microcalcifications (arrows) on the submucosal layer (or epithelium) (×200, hematoxylin and eosin stain).

**Figure 4:**
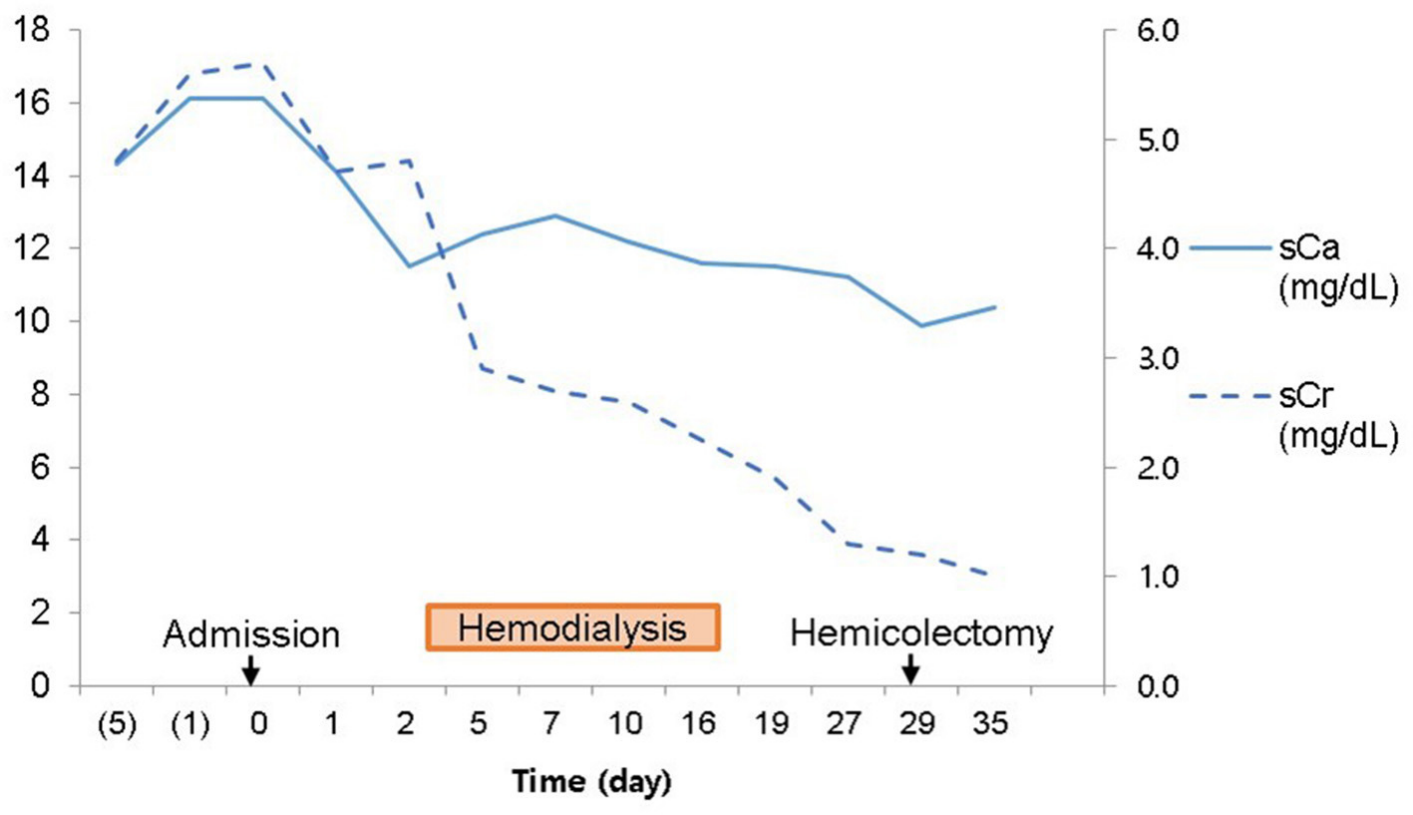
Clinical course of hypercalcemia and azotemia during admission.

We suspected another cause of hypercalcemia. The patient reported consuming 1,000 to 1,200 mL of milk daily with intermittent commercial herbal pills for 3 months. He took the isopropanol extract of Artemisia princeps for anorexia. He took mixed extract from Angelica, Chaenomeles, Ledebouriella, Dipsacus for arthralgia. He had even consumed milk continuously during admission. We advised withdrawal of milk and the herbal pills. On admission day 17, we ended hemodialysis because azotemia (sCr 1.9 mg/dL) and hypercalcemia (sCa 11.6 mg/dL) were resolving. Then, the patient underwent hemicolectomy for colon cancer on admission day 29. We found calcium deposits on colon biopsy (Figure 3). His sCa (10.4 mg/dL) and sCr (1.04 mg/dL) were normal at discharge on admission day 35 (Figure 4) and maintained normal values during follow-up for 1 year and until now at the outpatient clinic. Stomach calcifications persisted for 6 months (Figure 2B) and lung calcifications remained on bone scan after 1 year (Figure 2C).

## Discussion

MAS is not an uncommon disease and ranks as the third most common cause of hypercalcemia in hospitalized patients in the United States, after hyperparathyroidism and malignancy (6). The prevalence of MAS in the West was related to the Sippy therapy that is to drink milk with sodium bicarbonate and calcinate magnesia or bismuth subcarbonate for peptic ulcer (7, 8).

These days, nutritionists recommend consuming milk because it provides important nutrients, and the high protein and mineral content support growth. However, reports of MAS in Asians are rare. In the past Far East Asian didn't consume milk nor Sippy regimen as much as western. However, people in the Far East Asia nowadays tend to drink more milk and sometimes in excess, as seen in this patient. Recommended daily allowance (RDA) of calcium differs according to age, ethnicity, and nationality (9). RDA of calcium for adults is different between North American (1,000 mg to 1,200 mg) and Korean (700 to 800 mg) (10). The calcium content has been found to vary between 1,160 and 3,360 mg/L among the 26 different milk brands sold in Korea (11).

Patel et al. suggest to call MAS “calcium-alkali syndrome” because calcium and vitamin D intake is an important cause of this morbidity nowadays (12). The dose and duration of calcium intake that provoke MAS are variable. In one case, a 47-year-old woman taking calcium carbonate (1,000 mg) plus vitamin D (400 U) twice daily experienced MAS (3). A 26-year-old Caucasian woman ingesting 2,500–4,400 mg daily throughout pregnancy was also diagnosed with MAS (13). Another case described a 54-year old male who ingested about 11,270 mg of calcium equivalent (70 tablets of Tums, OTC antacid) and baking soda in 3 days (14). In the present case, the patient drank above 1,000 mL of milk per day for 3 months as a substitute for oral intake. We stopped him from drinking milk because he continued to drink milk even at the beginning of hospitalization.

Diagnosis of MAS is based on unexplained hypercalcemia, variable grade of renal injury, and metabolic alkalosis due to excessive calcium and absorbable alkali supplementation (3, 6). Investigation of calcium-containing drugs and diet is important for the diagnosis of MAS. A history of underlying disease requiring calcium or vitamin D supplementation and checking of prescription are helpful because patients might not know whether the components of their medications contain calcium or vitamin D. Since hypercalcemia has no characteristic symptoms and signs before aggravation, it tends to be recognized first in blood test. The blood test needs to include both 25-OH vitamin D and 1,25-(OH)2 vitamin D for evaluating the vitamin D-associated hypercalcemia if hypervitaminosis is suspected in the history taking (15). Genetic testing is becoming a routine part of the investigation of hypercalcemia where inherited or familial hyperparathyroidism is suspected (16). Oyster shells and several medications including aluminum hydroxide and magnesium were reported for the source of absorbable alkali in MAS (17). Although herbal medicines have never been reported as causative agents for alkali, this possibility cannot be definitely excluded. We need to pay more attention to its ingredients in patients taking herbal medicines. We were able to diagnose as MAS because this patient presented hypercalcemia with metabolic alkalosis and azotemia.

Nausea, vomiting, anorexia, distaste for milk, headache, dizziness, vertigo, apathy, and confusion are early signs of hypercalcemia. This patient had dizziness and anorexia for several months, but he did not visit the hospital. The cause of these symptoms was thought to be multifactorial, involving anemia and colon cancer as well as hypercalcemia and azotemia.

Renal calcium handling in MAS depends on a variety of factors, most but not all of which act to increase calcium reabsorption. Hypercalcemia itself can cause hypercalciuria. But renal injury due to hypercalcemia reduce the calcium filtration. And hypercalcemia activates the calcium-sensing receptor (CaSR) in the thick ascending limb of Henle and medullary collecting duct which cause hypercalciuria, natriuresis and diuresis. But volume depletion by diuresis decreases the glomerular filtration rate and calcium filtration. Metabolic alkalosis enhances calcium reabsorption (8). Our patient had hypercalciuria, probably due to hypercalcemia.

Three forms of MAS have been described: acute, subacute (Cope syndrome) and chronic (Burnett syndrome) type (18). Chronic hypercalcemia leads to metastatic calcifications due to the long disease duration. Renal calcinosis is also common. Other less common sites of metastatic calcification are periarticular tissue, subcutaneous tissue, central nervous system, liver, adrenal system, bone, lungs, breast, and stomach (3, 19). Calcified lesions are diagnosed via many methods like bone scan, ultrasonography, CT, PET-CT, and pathology. Bone scans provided important clues to diagnose chronic MAS in this patient. The most common sites of metastatic calcification on bone scan are the kidneys, lungs, gastric mucosa, and, more rarely, the heart, thyroid, liver, skeletal muscles, or blood vessels (20). Coolens et al. reported that the most common cause of pulmonary calcification on bone scan is hyperparathyroidism, and then, multiple myeloma, lymphoma, metastatic breast cancer and vitamin D intoxication are followed (21). This patient showed calcifications in the kidney, stomach, lung, and colon. In this patient, acute kidney injury improved within 2 weeks. Hypercalcemia improved within 1 month, but improvement of tissue calcification on bone scan took more than 1 year.

Treatment of MAS varies according to severity. First and the most important supportive care is suspending diets and drugs containing calcium and/or other alkali agents. Mild disease can be resolved by stopping supplementation. One simple medical treatment is large amounts of hydration with isotonic saline (1–2 L isotonic saline bolus, then 100–150 ml/hour, maintaining a urine output of 100 ml/hour). Bisphosphonates can be considered as an additional option or first-line treatment. However, some bisphosphonate agents like pamidronate or zolendronic acid can be nephrotoxic and this is not desirable for patients with a creatinine clearance below 30 ml/min (22). For the emergency management of hypercalcemia, calcitonin, dialysis and mithramycin can be used. More recently, also denosumab, a bone anti-resorptive agent used in the treatment of osteoporosis, has been used (16). Corticosteroids is used for the treatment of hypercalcemia due to hematologic malignancy, granulomatous disease, idiopathic hypercalcemia, and hypervitaminosis D. Cinacalcet, one of the calcimimetic, reduce calcium levels caused by primary hyperparathyroidism. Ketoconazole and hydroxychloroquine are used for hypercalcemia related to sarcoidosis. And hemodialysis with low or zero calcium dialysate and continuous renal replacement therapy are useful in patients with severe hypercalcemia and azotemia like this patient (1).

Microcalcification associated tumor is well-described in the breast cancer. It is associated with HER2 overexpression and poor prognostic factor of survival and recurrence (23). The calcium sensing receptor is associated with calcification in the prostate cancer (24). Hypercalcemia can facilitate the proliferation and metastasis of stomach and colon cancer, and it is a marker of metastasis (25). Because this patient had colon cancer, we had to find additional evidence to exclude malignancy-related hypercalcemia. There are few mechanisms of hypercalcemia induction in malignancy. First of all, PTHrP is involved in nearly 80% of these mechanisms (26). However, PTHrP was not elevated in this case. Non-PTHrP pathways consist of osteolytic metastasis, overproduction of 1,25 vitamin D, and parathyroid carcinoma and ectopic production of PTH. There were no osteolytic metastatic lesions on PET-CT and bone scans in this patient, nor did he exhibit elevated 25-OH vitamin D or parathyroid carcinoma with ectopic production of PTH. Therefore, we concluded that there was no evidence for malignancy-related hypercalcemia.

## Conclusions

Chronic MAS is rare. We successfully treated acute kidney injury due to Burnett syndrome in a patient with colon cancer despite missing the patient's history of excessive milk consumption at presentation. The importance of precise history taking in hypercalcemia cannot be overstated. We are likely to suspect paraneoplastic syndrome as the cause of hypercalcemia in patients with cancer because of its high frequency. However, in order to avoid invasive tests and facilitate timely diagnosis, excessive calcium intake should not be overlooked.

## Data Availability Statement

The original contributions presented in the study are included in the article/supplementary material, further inquiries can be directed to the corresponding author.

## Ethics Statement

The studies involving human participants were reviewed and approved by Institutional Review Board at Gyeongsang National University Hospital, approval number: GNUH 2021-10-014. The patients/participants provided their written informed consent to participate in this study.

## Author Contributions

HL, SY, and B-HC drafted the manuscript, contributed to the case collection, provided Figures 1–4, and approved the final manuscript as submitted. SL, SJ, HJ, and S-HC contributed to the study design and approved the final manuscript as submitted. H-JK provided major treatment on the patient while admitted and outpatient clinic and approved the final manuscript as submitted. All authors contributed to the article and approved the submitted version.

## Conflict of Interest

The authors declare that the research was conducted in the absence of any commercial or financial relationships that could be construed as a potential conflict of interest.

## Publisher's Note

All claims expressed in this article are solely those of the authors and do not necessarily represent those of their affiliated organizations, or those of the publisher, the editors and the reviewers. Any product that may be evaluated in this article, or claim that may be made by its manufacturer, is not guaranteed or endorsed by the publisher.
